# Antioxidant and Anti-Hepatitis C Viral Activities of Commercial Milk Thistle Food Supplements

**DOI:** 10.3390/antiox2010023

**Published:** 2013-02-06

**Authors:** Kevin Anthony, Gitanjali Subramanya, Susan Uprichard, Faiza Hammouda, Mahmoud Saleh

**Affiliations:** 1 Department of Chemistry, Texas Southern University, Houston, TX 77004, USA; E-Mail: anthonykp@tsu.edu; 2 Department of Medicine, University of Illinois Chicago, Chicago, IL 60612, USA; E-Mails: gsubramanya@luc.edu (G.S.); suprichard@lumc.edu (S.U.); 3 Department of Phytochemistry, National Research Center, Dokki 12311, Cairo, Egypt; E-Mail: fmhammouda@yahoo.com

**Keywords:** *Silybum marianum*, radical scavenger, food supplement, over the counter drugs, hepatitis C virus

## Abstract

Milk thistle dietary supplements that contain silymarin are widely marketed and used in the USA and other countries for liver enhancement and recovery. More recently, silymarin has also been identified as a possible antiviral for the treatment of hepatitis C virus (HCV) infection. To assess different brands of commercially sold silymarin, 45 products were collected from local stores and analyzed for their silymarin content, antioxidant activities, and antiviral activity against HCV. Antioxidant activity was measured as radical scavenging activity using DPPH and by estimating their antioxidant capacity as trolox equivalent. Anti-HCV activity was measured in an HCV genotype 1b replication inhibition assay. Samples were found to vary widely in their silymarin content, with some samples having none or very low concentrations while silymarin represented higher than 80% of other samples. Both antioxidant and anti-HCV activity correlated with the overall level of silymarin.

## 1. Introduction

Over-the-counter nutritional or dietary supplements are becoming extremely popular in the United States, Europe and many other countries. As defined by the USA Food and Drug Administration (FDA), a dietary supplement is a product taken by mouth that contains a “dietary ingredient,” which can be vitamin, mineral, herb, amino acid, enzyme, or metabolite. Traditional medicines, including medicinal herbs and their preparations, are used as part of the primary health care for 70%–95% of the population in the developing world, while over 70% of the population in developed nations use some form of complementary/alternative medicines [1]. Nearly 50% of older adults regularly use dietary aids [2]. As a result, one recent estimate of the global market for traditional medicines was $83 billion annually with the expectation that this will grow considerably in the coming years [3].

**Figure 1:**
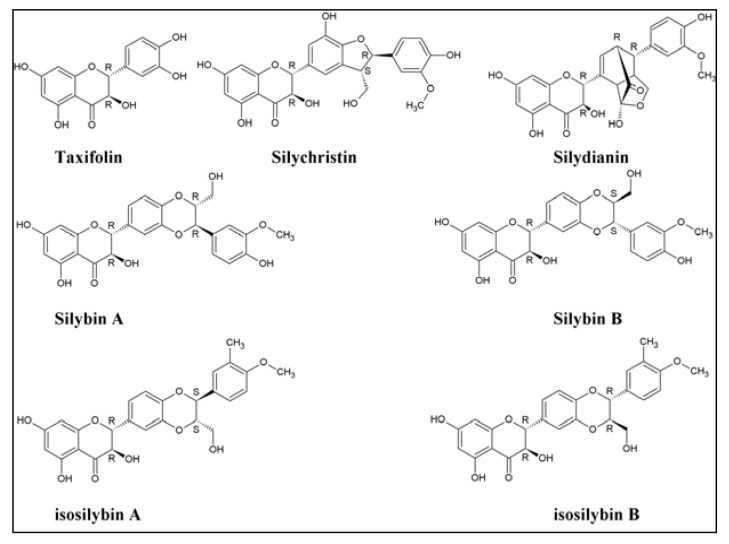
Chemical structure of the major constituents of silymarin.

One of the products that has gained popularity in recent years is milk thistle seed extract, also known as silymarin, which is sold under many different brand names. Silymarin is isolated from the milk thistle plant *Silybum marianum* of the family Asteraceae. The product is advertised as a hepatoprotective, antioxidant, antiradical, and free radical scavenging food supplement and has been used widely for centuries for the protection of the liver from toxic substances, treating liver damage and for the therapy of hepatitis and cirrhosis [4,5,6,7]. In addition to its antioxidant properties, it has been reported to have high anti-tumor promoting activity [8] and has been linked to the prevention of skin carcinogenesis [9]. Recent studies have also reported that silymarin is an effective antiviral treatment for hepatitis C virus (HCV) [10,11,12,13,14,15,16,17]. Silymarin is a mixture of seven major compounds: taxifolin, silychristin, silydianin, silybin A, silybin B, isosilybin A and isosilybin B [18,19]. The chemical structures of the seven main active constituents of silymarin are shown in Figure 1.

The reason we undertook the current study is that complexity of the silymarin product combined with its unregulated manufacturing process has made it difficult to judge the role of silymarin in the treatment of chronic liver diseases. This has been further compounded by the poor documentation of the ingredients in these products, the source of the silymarin or the specific extraction processes used. For example, harvesting herb plants in different geological regions and seasons is well known to affecting the quantities of the chemical components of the plants and potentially the efficacy of the extracts [20,21,22]. In the absence of specific criteria or guidelines for the judging the quality of silymarin extracts, it has been difficult to interpret the majority of previous clinical efficacy studies, reconcile what may seem to be conflicting results among different studies or determine the individual active components [23,24]. Thus to begin to compare the silymarin content and representative biological activities of different commercial milk thistle sources, 45 milk thistle commercial preparations were collected. High performance liquid chromatography/mass spectrometry (LC/MS) was used to empirically determine in parallel the total amounts of silymarin, likewise, antioxidant and anti-HCV activities were measured side-by-side in standardized assays.

## 2. Experimental Section

### 2.1. Chemicals and Reagents

All solvents used for HPLC and MS analyses were of chromatographic grade, formic acid and, dimethyl sulfoxide (DMSO) were purchased from VWR International Co. (Sugar Land, TX, USA). Technical silymarin (>96% pure) and 1,1-diphenyl-2-picrylhydrazyl (DPPH) were purchased from Sigma Aldrich Inc. (Atlanta, GA, USA). All commercial over-the-counter milk thistle food supplements samples were obtained from USA and International markets. Samples identification numbers, sources and brand names are shown in (Table 1).

**Table 1:** Commercial milk thistle samples.

Sample ID/Description/Sources & Average weight of each tablet		Sample ID/Description/Sources & Average weight of each tablet	
1	Swanson Superior Herbs^®^	24	Nature’s Bounty^®^/Natural Whole Herbs
	Milk Thistle 80% Silymarin/USA (0.5504 g/tablet)		Milk Thistle 1000 mg/USA (0.3939 g/tablet)
2	Ortho Molecular Products^®^	25	Legalon^®^ 140 Silymarin Egypt/Germany (0.3939 g/tablet)
	Silymarin Forte/USA (0.4502 g/tablet)		
3	Metabolic Response Modifiers^®^	26	Hepatic Forte^®^
	Silymarin with Bio Sorb/USA (0.4975 g/tablet)		Silymarin/Egypt (0.7944 g/tablet)
4	Advance Physician Formulas^®^ (0.3319 g/tablet)	27	Levatech^®^
	Milk Thistle (Standardize Silymarin 80%)/USA		Silymarin/Egypt (0.4569 g/tablet)
5	Pure Encapsulations^®^ (0.2430 g/tablet)	28	Livit^®^ (1.3158 g/tablet)
	Silymarin Milk Thistle Extract/USA		Liver support, Soft gelatin capsules/Egypt
6	Thorne Research T.A.P.S^®^	29	Liverin^®^ (0.6589 g/tablet)
	Dietary Supplement/USA (0.6789 g/tablet)		Improvement of liver function/Egypt
7	Metagenics^®^	30	Levatone^®^
	Silymarin 80/USA (0.2450 g/tablet)		Food Supplement/Egypt (1.2484 g/tablet)
8	Himalaya Liver Care^®^	31	Liver Albumin Plus^®^ (0.9610 g/tablet)
	Liv.52/USA (0.3768 g/tablet)		Dietary Supplement/Egypt
9	Jarrow Formulas^®^/USA (0.2786 g/tablet)	32	Hipamax Plus^®^ (1.5340 g/tablet)
	Milk Thistle (Standardize Silymarin Extract 30:1)		Dietary Supplement/Egypt
10	Metabolic Maintenance^®^	33	SEDICO^®^ (13597 g/tablet)
	Silymarin/USA (0.5696 g/tablet)		Silymarin Plus, Dietary Supplement/Egypt
11	Life Extension^®^ (0.9040 g/tablet)	34	Hepaticum^®^ (0.4228 g/tablet)
	Mega Silymarin with isosilybin B/USA		Cyclodextrin enhanced formula/Egypt
12	Purintin’s Pride^®^	35	Silipex^®^ (0.3351 g/tablet)
	Silymarin Milk Thistle/USA (0.3466 g/tablet)		Dietary Supplement /Egypt
13	Natural Wellness^®^/USA (0.5063 g/tablet) Maximum Milk Thistle, Silybin Phytosome 240 mg	36	MEPACURE^®^ (0.3907 g/tablet)
			Liver support /Egypt
14	Enzymatic Therapy^®^	37	Hepanox^®^ Cap. (1.3371 g/tablet)
	Super Milk Thistle/USA (0.3262 g/tablet)		Napha food support/Egypt
15	Advanced Beta Glucon Therapy^®^ (0.4867 g/tablet)	38	SELECTIVAL^®^ (1.2695 g/tablet)
	Bio-Silymarin, Aloha Medicinal Inc./USA		Dietary Supplement/Egypt
16	Futurebiotics^®^	39	Ursoplus^®^ MINAPHARM
	Silymarin Plus/USA (1.0514 g/tablet)		Silymarin 70%/Egypt (0.5625 g/tablet)
17	Planetary Herbals^®^ (0.7060 g/tablet)	40	Leaglon^®^ 70 Silymarin/Egypt/Germany (0.4259 g/tablet)
	Full Spectrum Silymarin 80™/USA		
18	Wonder Laboratories^®^ Advanced B-12 Sublingual/USA (0.3443 g/tablet)	41	Hepamarin^®^ 140mg
			Hepatoprotective/Egypt (0.3124 g/tablet)
19	21st Century^®^ (0.4508 g/tablet)	42	Trade Mark^®^ (0.0518 g/tablet)
	200 count Milk Thistle Extract/USA		Biphenyldicarboxylate/China/Egypt
20	Source Naturals^®^	43	MEPASIL^®^ (0.5075 g/tablet)
	Silymarin Plus/USA (0.9847 g/tablet)		Silymarin, Liver support/Egypt
21	Now^®^	44	MARIAGON^®^ (0.4775 g/tablet)
	Silymarin 100 V caps/USA (0.5090 g/tablet)		Hepatoprotective/Egypt
22	Good’N Natural^®^ Milk Thistle Extract 250 mg/USA(0.6765 g/tablet)	45	Hepato-Forte^®^ (1.2624 g/tablet)
			Liver Supplement/Egypt
23	TwinLab^®^		
	Silymarin/USA (0.1610 g/tablet)		

### 2.2. Cells

The Clone B HCV genotype 1b sub-genomic (sg1b) replicon Huh7 cells, which constitutively replicate a subgenomic HCV RNA in their cytoplasm, were obtained from C.M. Rice (Rockefeller University, NY, USA) through the NIH AIDS Research and Reference Reagent Program and have been described previously [25]. Cells were cultured in complete Dulbecco’s modified Eagle’s medium (cDMEM) (Hyclone, Logan, UT, USA) supplemented with 10% fetal bovine serum (FBS) (Hyclone), 100 units/mL penicillin, 100 mg/mL streptomycin, and 2 mM l-glutamine (Gibco Invitrogen, Carlsbad, CA, USA) as previously described [26].

### 2.3. Preparation of Samples

Ten tablets of each commercial sample were randomly taken, crushed and homogenized. Weight of each 10 tablets was recorded and is presented for each brand sample (Table 1). For chromatography and mass spectrometry analysis: 20 mg of each crushed product were extracted in 5 mL of methanol (3 replicate each). For all bioassay evaluations a second batch of 20 mg of each product were separately extracted in 5 mL of DMSO (3 replicate). Extractions were performed in 10 mL sealed tubes at room temperature rotated constantly using a Labnet Labroller II (Optics Planet Inc., 3150 Commercial Avenue Northbrook, IL, USA), at maximum speed for 24 h. Extracts were then filtered and stored in the refrigerator. External calibrated standards were made under the same condition for technical silymarin (Sigma Products).

### 2.4. Determination of Total Silymarin

High Performance Liquid chromatography and Mass Detection HPLC/MS were used to determine the chemical composition of each the commercial products as previously described by us [27]. HPLC of silymarin and commercial samples was performed on Agilent 1100 HPLC/MSD VL using Phenomenex Kinetic 2.6 μ C_18_ 100 A 100 × 4.16 mm column with electrospray (ES) ionization. Methanol, water, and formic acid (90:10:1) was used as mobile phase A and 0.1% formic acid for mobile phase B at a gradient flow rate of 0.5 mL/min. Solvent A = 55% 0.1 formic acid, solvent B = 45% 90:10:1 MeOH:H_2_O:Formic acid. Starting at time 0, 45% B, at 15 min, increase to 65% B, at 15.5 min decrease to 45% B and hold at 45% B for 5 min run end 20.5 min and diode array detection at 288 nm. Mass Spectroscopy was performed using single ion monitoring in the positive ESI mode for ions of *m/z* 327 (M + Na) for taxifolin and *m/z* 505 (M + Na) for all other isomers. Mass detection conditions were: quasi molecular ions dwell time of 294 ms, nitrogen was used both as drying gas and nebulizing gas at flow rates of 12 L/min and 35 (psig). The temperature of the drying gas was set to 350 °C. Data collection was handled using Chemstation V. B.04.02. All samples (45 × 3 replicate) were analyzed by injecting 5 μL of sample in methanol and the analysis was repeated three times to calculate the average and standard deviations.

### 2.5. Free Radical-Scavenging Activity: DPPH Test

Free radical-scavenging activity of each commercial sample was carried out using the DPPH scavenging method [28]. The antioxidant activity was carried out using Perkin Elmer Victor 4X micro plate reader performed in a 96 well plate using a total volume of 200 μL methanol containing 0.004 μg DPPH and samples aliquots at a series of concentrations of 1, 10, 20, 40, 60, 80, 200, 400, 800 and 2000 μg/mL. The test was repeated at all concentration of each sample in triplicate. DPPH solutions at the same concentration without the tested samples were used as control. Each sample, as well as each control was analyzed in triplicates. After filling the well plates, they were incubated in the dark with continuous shaking for 30 min followed by reading the absorbance at 520 nm. The free radical scavenging activity of each solution was then calculated as percent inhibition according to the following equation:

% inhibition = 100 × (A_blank_ − A_sample_)/A_blank_ (1)

where A_sample_ is the absorbance of the sample and A_blank_ is the absorbance of the blank. Inhibition % was plotted against concentration and the EC_50_ was calculated graphically.

### 2.6. Trolox-Equivalent Antioxidant Capacity Assay

Trolox-equivalent antioxidant capacity (TEAC) of the commercial silymarin samples was carried out using the procedure from Antioxidant Assay Kit item No. 709001 from Cayman Chemical Company1180 E. Ellsworth Rd. Ann Arbor, MI, USA. The 45 commercial silymarin samples were prepared by removing 100 μL of the stock preparation (20mg commercial silymarin/5 mL of DMSO) and adding it to 400 μL HPLC grade water. On a 96 well plate, 10 μL of this preparation was removed and added to 10 μL of metmyoglobin, 150 μL of chromogen and 40 μL of hydrogen peroxide mixture for a total of 210 μL in each well. The plate was covered and place on a shaker for five minutes and read at 750 nm on a Perkin Elmer Victor X4 2030 Multilabel Reader (710 Bridgeport Avenue Shelton, CT, USA). The absorbance was plotted as a function of the final Trolox concentration (mM) according to the assay.

Antioxidant (mM) = Sample absorbance − (Y − intercept)/Slope × Dilution (2)

### 2.7. Anti HCV Bioassay

Clone B sg1b cells were seeded in 96-well BIOCoat culture plates (BD Biosciences) at a density of 8 × 10^3^ cells/well in cDMEM. Upon reaching 90%–95% confluence, media was replaced with 200 μL cDMEM supplemented with 1% DMSO (Sigma) and cells were cultured for an additional 20 days, replacing medium every 2 days as previously described [29,30,31]. After these 20 days of culturing, testing of the silymarin samples was initiated in parallel cultures of Clone B replicon cells. Cells were treated with the individual silymarin samples at the indicated doses or diluents (DMSO) control. On days 2, 4 and 6 post-silymarin treatment initiation medium was collected from culture plates and stored for cytotoxicity analysis as described below. On day 6 post-silymarin treatment, cells were lysed in 200 μL 1X Nucleic Acid Purification lysis solution (Applied Biosystems, Foster City, CA, USA) and immediately frozen (−80 °C). Real-time quantitative PCR (RTqPCR) analysis was performed as described below to quantify intracellular HCV RNA levels.

### 2.8. Cytotoxicity Assay

Silymarin-mediated cellular toxicity was determined using the Toxilight Bioassay Kit (Lonza, Walkersville, MD, USA), a bioluminescence-based assay which measures adenylate kinase (AK) released from damaged cells, as per the manufacturer’s instructions. 

### 2.9. RNA Isolation and RTqPCR Analysis

Total cellular RNA was isolated using a 1X Nucleic Acid Purification Lysis Solution (Applied Biosystems, Foster City, CA, USA) and purified using an ABI PRISM™ 6100 Nucleic Acid PrepStation (Applied Biosystems), as per the manufacturer’s instructions. One μg of purified RNA was used for cDNA synthesis TaqMan reverse transcription reagents (Applied Biosystems) and FastStart Universal SYBR Green master mix (Roche Applied Sciences, Indianapolis, IN, USA), using an Applied Biosystems 7300 real-time thermocycler (Applied Biosystems). Thermal cycling consisted of an initial 10 min denaturation step at 95 °C followed by 40 cycles of denaturation (15 s at 95 °C) and annealing/extension (1 min at 60 °C). HCV JFH-1 and GAPDH transcript levels were determined relative to a standard curve comprised of serial dilutions of plasmid containing the JFH-1 HCV cDNA or the human GAPDH gene, respectively. The PCR primers used to detect GAPDH and HCV were: human GAPDH5′-GAAGGTGAAGGTCGGAGTC-3′ (sense) and 5′-AAGATGGTGATGGGATTTC-3′ (anti-sense) and JFH-1 HCV 5′-TCTGCGGAACCGGTGAGTA-3′ (sense) and 5′-TCAGGCAGTACCACAAGGC-3′ (anti-sense).

### 2.10. Statistical Analysis

Data was entered in SPSS Statistics [32] and analyzed using Pearson and Spearman nonparametric correlation analysis with two-tailed significance determined.

## 3. Results and Discussion

### 3.1. Determination of Total Silymarin

**Figure 2:**
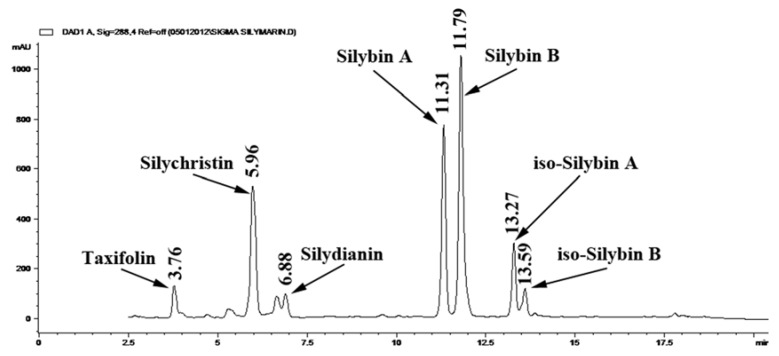
HPLC chromatograms of silymarin.

Because the harvesting, extraction, manufacturing, and quantification techniques used for generating commercially available for the treatment of chronic liver diseases is unregulated, it is difficult to directly compare different commercial sources of milk thistle and interpret the many studies that have reported various levels of biological activities. Hence, we sought to compare the actual content of silymarin among 45 different milk thistle products. Quantitative analysis of total silymarin in all of the selected commercial samples was performed with 3 replicate extracts and three analytical measurements. Total silymarin was calculated based on the sum of all of the major silymarin constituents as shown in Figure 2. Average concentrations and standard deviation of each sample are shown in Table 2 as mg per gram of tablet.

**Table 2:** Total silymarin concentration, antioxidant and anti-HCV activities in the tested samples.

ID	Total Silymarin mg/gram tablet	Antioxidant Activity		Anti-HCV activity
		DPPH	Trolox Equivalent mmol/g	% inhibition
		EC_50_μg/mL		(100 μg of tablet/mL)
1	366.7 ± 0.8	1.08 ± 0.02	9.0 ± 0.1	84 ± 8
2	478.5 ± 1.1	0.98 ± 0.02	9.3 ± 0.3	50 ± 4
3	107.0 ± 0.5	2.97 ± 0.02	5.3 ± 0.2	45 ± 21
4	467.4 ± 1.1	1.00 ± 0.00	8.9 ± 0.5	60 ± 20
5	849.9 ± 0.8	0.45 ± 0.00	9.0 ± 0.7	85 ± 5
6	57.8 ± 0.9	0.98 ± 0.13	4.1 ± 2.2	16 ± 16
7	326.0 ± 1.0	2.00 ± 0.00	7.7 ± 0.8	62 ± 14
8	Not detected	0.20 ± 0.00	9.5 ± 0.5	24 ± 23
9	467.8 ± 1.9	1.03 ± 0.02	8.7 ± 0.4	39 ± 7
10	568.3 ± 1.7	0.92 ± 0.02	9.2 ± 0.4	70 ± 8
11	848.7 ± 1.1	0.25 ± 0.01	9.1 ± 0.5	76 ± 11
12	67.7 ± 0.4	4.40 ± 0.00	3.4 ± 0.5	30 ± 5
13	162.4 ± 0.5	6.13 ± 0.09	4.8 ± 0.7	38 ± 22
14	75.0 ± 0.2	1.20 ± 0.00	5.8 ± 0.9	18 ± 2
15	685.1 ± 3.3	0.30 ± 0.00	9.4 ± 0.4	72 ± 2
16	81.2 ± 0.3	2.37 ± 0.12	4.0 ± 0.3	39 ± 25
17	314.9 ± 0.5	1.08 ± 0.02	8.3 ± 0.5	72 ± 1
18	82.4 ± 0.3	3.03 ± 0.05	6.6 ± 1.4	20 ± 17
19	109.5 ± 0.9	2.17 ± 0.05	7.6 ± 1.5	81 ± 4
20	248.5 ± 0.1	1.00 ± 0.00	7.5 ± 0.6	88 ± 8
21	341.9 ± 0.5	0.90 ± 0.00	8.9 ± 0.5	81 ± 4
22	276.8 ± 1.1	1.20 ± 0.00	7.7 ± 0.1	80 ± 12
23	408.4 ± 0.4	1.10 ± 0.00	9.2 ± 0.1	81 ± 20
24	72.6 ± 0.4	4.23 ± 0.12	4.0 ± 0.8	Not tested
25	522.8 ± 0.9	0.95 ± 0.04	9.0 ± 0.2	62 ± 2
26	88.6 ± 0.3	3.13 ± 0.19	1.9 ± 1.2	35 ± 12
27	260.8 ± 0.4	1.20 ± 0.08	7.4 ± 0.3	51 ± 14
28	145.1 ± 0.1	2.80 ± 0.49	3.8 ± 1.0	17 ± 11
29	Not detected	>10.00	0.0	42 ± 10
30	111.9 ± 0.1	2.27 ± 0.25	3.9 ± 1.4	10 ± 14
31	132.8 ± 0.1	4.08 ± 0.23	2.6 ± 1.1	14 ± 9
32	113.2 ± 0.3	3.87 ± 0.57	1.7 ± 0.9	47 ± 36
33	177.1 ± 0.1	0.31 ± 0.00	4.2 ± 0.7	24 ± 7
34	450.2 ± 0.6	1.10 ± 0.07	8.0 ± 1.1	35 ± 23
35	339.5 ± 0.2	0.95 ± 0.04	7.5 ± 1.0	48 ± 16
36	83.6 ± 0.5	2.17 ± 0.05	5.3 ± 0.8	26 ± 6
37	113.2 ± 0.1	1.18 ± 0.44	2.8 ± 0.9	33 ± 10
38	227.6 ± 0.1	2.07 ± 0.09	5.4 ± 0.7	37 ± 10
39	226.3 ± 0.3	1.67 ± 0.26	5.8 ± 0.8	46 ± 20
40	274.3 ± 0.5	2.13 ± 0.05	7.4 ± 0.5	46 ± 19
41	383.8 ± 0.3	1.03 ± 0.05	8.2 ± 0.8	42 ± 9
42	Not detected	>10.00	0.0	15 ± 21
43	346.9 ± 0.9	1.13 ± 0.09	7.6 ± 1.3	45 ± 9
44	303.8 ± 0.6	1.15 ± 0.04	7.4 ± 0.9	21 ± 3
45	Not detected	>10.00	0.0	28 ± 2

### 3.2. Free Radicals Scavenging Activity

**Figure 3:**
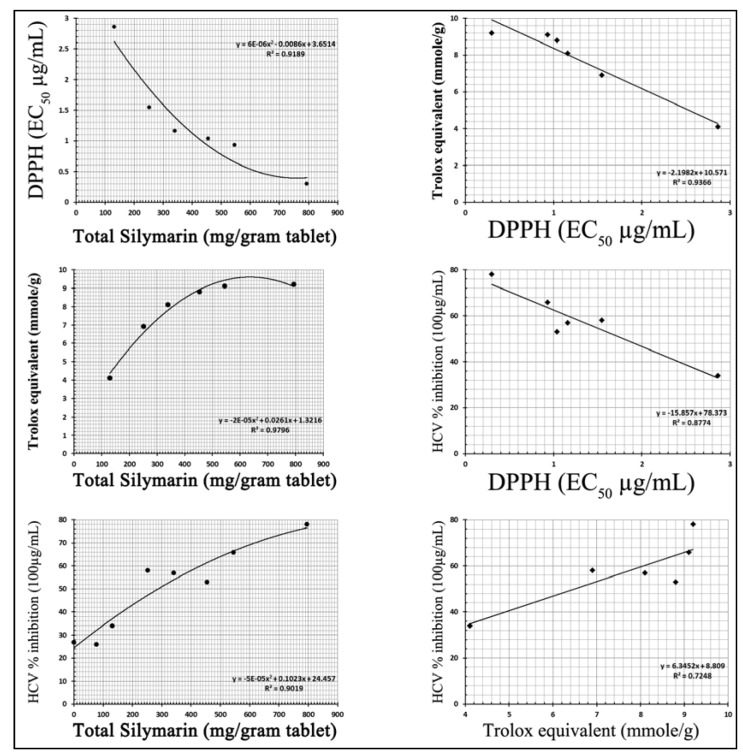
Correlation between total amounts of silymarin in the tested samples verses their Trolox equivalent antioxidant capacity, DPPH radical scavenging EC_50_ and anti HCV activities.

To determine whether the relative total silymarin content measured in each product corresponds with different biological activities reported for silymarin, we first assayed anti-radial activity by a (DPPH) assay. Hence, the change in absorbance produced by reduced DPPH was initially used to evaluate the ability of the silymarin samples to act as free radical scavengers with the lower the value of EC_50_ indicating higher anti-radical power. Thirteen samples showed an EC_50_ of less than 1 μg/mL, 14 samples were shown to have an EC_50_ of 1 to less than 2 μg/mL, 11 samples had an EC_50_ greater than 2 but less than 4 μg/mL, and 7 samples of had an EC_50_ greater than 4 μg/mL (Table 2). Consistent with the literature indicating that silymarin can act as a as free radical scavengers and consistent with the conclusion that the majority of silymarin detected in these specific products appears to be present in an active form, the EC_50_ in all cases was generally found to be related to the total amount of silymarin in each tablet (*R*^2^ = 0.9189; Figure 3). Of course, this correlation was not perfect raising the question of whether different harvest, extraction and/or storage conditions might alter the inherent specific activity of the silymarin found in different products.

### 3.3. Total Antioxidant Capacity as Trolox Equivalent (TEAC)

Analogously, the silymarin samples were also assayed for their total antioxidant capacity as equivalent to trolox (TEAC). TEAC values of all of the commercial samples varied greatly with samples showing no antioxidant capacity to samples with 9 or higher mmols trolox equivalent/g (Table 2). While again outliers were present, when the average concentration of silymarin in the samples plotted against their average TEAC (Figure 3), a correlation was apparent (*R*^2^ = 0.9796). Notably, the DPPH and TEAC assays gave very similar results: lowest DPPH EC_50_ values correlated with the highest TEAC values, while highest DPPH EC_50_ values correlated with the lowest TEAC values suggesting that perhaps these activities are closely related (Table 2, Figure 3). However, the DPPH test showed a broader range of linearity, especially at the highest silymarin concentrations (Figure 3).

### 3.4. Anti HCV Activity

As previous studies have reported the ability of silymarin to inhibit replication of HCV sg1b replicons, anti-HCV activity of the individual silymarin samples was assessed using sg1b replicon cells. Based on initial dosing and toxicity screening (data not shown) each product sample was screen at three non-toxic doses (100, 25, 6.25 μg of tablet/mL). On 6 post-treatment culture media was collected for confirmatory toxicity analysis using an AK release cellular membrane integrity assay and total RNA was isolated from cell lysates for RT-qPCR analysis of HCV and cellular GAPDH. HCV RNA levels were normalized to GAPDH and then compared to the average HCV RNA level present in replicate mock treated cultures. As a positive control for HCV inhibition, parallel cultures were treated with 100 U/mL interferon-α. Additionally, we included treatment with the intravenous clinical Silibin formulation (SIL; Legalon) that has repeatedly demonstrated strong anti-HCV activity in patients. The percent inhibition achieved at the highest tablet equivalent (100 μg/mL) is shown in Table 2. As expected based on previous identical studies [30,31], 100 U/mL interferon-α reduce intracellular HCV sg1b RNA levels. Consistent with clinical data, the control also reduced intracellular HCV sg1b RNA levels, but to a lesser extent [30,31]. Again as observed for the free radical scavenging and antioxidant activity of the samples, the degree of HCV inhibition correlated with the relative level of total silymarin detected in each product (correlation coefficient = 0.650; *p* = 0.01). Interestingly, the HCV antiviral activity of the products did not correspond as well as with the DPPH and TEAC suggesting that the antiviral activity of silymarin may to some extent is distinct from the more closely related free radical scavenging and antioxidant activity and/or perhaps that different isomers of silymarin may be involved.

## 4. Conclusions

Our findings on different commercial preparations of silymarin are significant in light of the fact that oxidative stress is a secondary effect of many human diseases [33] and chronic HCV infection is a leading cause of liver disease worldwide. As such, consumption of antioxidant-containing foods can potentially reduce oxidative damage to cells and specifically reduce HCV-associated disease. Notably, because malnutrition depresses cellular immune function, consumption of these antioxidant-containing plants could have the general effect of protecting the immune system of malnourished individuals. This is relevant not only to HCV infection [34,35,36], but available data indicate that antioxidants are deficient in HIV infected populations due to increased utilization of antioxidant micro-nutrients [36] and observational studies suggest that increased intake of antioxidants may delay progression of HIV infection to AIDS [37,38].

Importantly however, our study documents that the different commercial sources tested varied greatly in overall silymarin While both anti-oxidant and anti-HCV activity exhibited some degree of correlation with silymarin levels suggesting that in the majority of products the silymarin present exhibited comparable levels of biological activity, we did note outliers for which this correlation was less apparent. There are several possible, non-mutually exclusive, explanation for these outliers: (1) Many of these products consist of a mixture of multiple extracts and/vitamins that also may contribute some biological activity in our assays. Thus, the absence of high levels of silymarin may not mean a sample is void of liver-related protective activities. (2) The potency of silymarin can vary from season-to-season, from one region to another, and/or be affected by specific extraction and storage conditions. (3) The ratio of the seven different silymarin isomers often can vary in different extracts depending on extraction methods and the plants used. Notably the ratio of the different silymarin isomers did vary among the various products, but in general the level of the different isomers was highly correlative making it difficult to determine if particular individual isomers were responsible to the various activities assayed in this limited sample set. Although preliminarily it appears that measurement of taxifolin concentrations in silymarin products may be an effective way of measuring the antioxidant potency of products from different suppliers, further analysis is required to conclusive determined which specific isomer(s) are responsible for the different therapeutic activities reported. Such future efforts are warranted as such insight could potentially, aid in the development of more potent silymarin formulations and inform regarding if specific isomer quantification could be used to standardize silymarin product activities.
